# Oral health-related quality of life after prosthetic rehabilitation: a longitudinal study with the OHIP questionnaire

**DOI:** 10.1186/s12955-015-0289-2

**Published:** 2015-07-10

**Authors:** Ágnes Jenei, János Sándor, Csaba Hegedűs, Kinga Bágyi, László Nagy, Csongor Kiss, Gyula Szabó, Ildikó J. Márton

**Affiliations:** Department of Restorative Dentistry, Faculty of Dentistry, University of Debrecen, 98 Nagyerdei Krt, Debrecen, H-4032 Hungary; Faculty of Public Health, University of Debrecen, 26 Kassai Street, Debrecen, H-4028 Hungary; Department of Prosthetic Dentistry, Faculty of Dentistry, University of Debrecen, 98 Nagyerdei Krt, Debrecen, H-4032 Hungary; Division of Pediatric Hemato-oncology, Department of Pediatrics, University of Debrecen, 98 Nagyerdei Krt, Debrecen, H-4032 Hungary; Division of Prosthodontics, Department of Dentistry, Oral and Maxillofacial Surgery, University of Pécs Medical School, 5 Dischka Győző Str, Pécs, H-7621 Hungary

**Keywords:** Quality of life, Prosthodontics, Oral health, Oral Health Impact Profile (OHIP) questionnaire

## Abstract

**Background:**

Aspects of oral health related quality of life (OHRQoL) attracted an increased attention recently.

**Objective:**

The aim of the study was to assess self-reported oral health related quality of life (OHRQoL) among patients requiring prosthetic rehabilitation and to determine the rate of improvement 1 month and 6–12 months after therapy. In addition, effect of age, gender, oral health indicators and denture types before treatment were assessed on OHRQoL as evaluated and reported by the patients.

**Methods:**

Hungarian version of OHIP-49 (OHIP-49-H) questionnaire was completed before oral rehabilitation (T0-phase) by 389 patients undergoing prosthetic replacement. After 1 month (T1-phase) and 6–12 months (T2-phase) recall periods 235 and 92 patients completed the questionnaire. The median interquartile range (IQR) values of the total OHIP-49-H score were calculated for T0-, T1- and T2-phases. Reliability of the questionnaire was checked by Cronbach’s statistics. Age, gender, oral health indicators and denture types of patients before and after treatment were recorded and treatment-associated changes in OHRQoL were evaluated.

**Results:**

The study demonstrated the excellent reliability and internal consistency of OHIP-49-H by a high and narrow range of Cronbach’s alpha value (0.81-0.93). A median OHIP-49-H score of 52; IQR = 25-83 demonstrated a poor OHRQoL on first admission. Decreasing median total OHIP-49-H scores 1 month (24; IQR = 9-51; *p* < 0.001) and 6–12 months (20; IQR = 7-37; *p* = 0,055) after therapy indicated an improvement of OHRQoL. Patients’ age and CPI value assessed before treatment proved to be significant factors of OHRQoL.

**Conclusions:**

Here we presented representative data about self-assessed OHRQoL of patients requiring prosthetic treatment from Hungary using OHIP-49-H questionnaire. The results demonstrated that the restoration of oral health was associated with an improvement in patients’ OHRQoL. According to the demographical and T0 phase clinical status, the treatment was more effective in the respect of OHIP-49-H score improvement among females (than among males), among younger (than among more aged), and among patients with more serious CPI assessed at T0. The type of prosthetic interventions did not exert a significant effect on total OHIP-49-H score, suggesting that the improvement in OHRQoL is independent from the type of denture applied.

## Background

Aspects of oral health related quality of life (OHRQoL) attracted an increased attention recently. The standard definition of health is determined briefly as freedom from disease, defect, or pain, according to the more precise definition of the World Health Organization in 1948, “health is a complete state of physical, mental, and social well-being, and not just the absence of infirmity” [1]. It has been accepted that the objective component of oral health (physical indicators) and the subjective component (patients’ perception of oral conditions) are complementary and cannot be separated in clinical practice [2].

There are several tools to assess OHRQoL. One of the most accepted measurement instrument is the internationally used Oral Health Impact Profile (OHIP) questionnaire [3]. In line with local adaptation, the original OHIP questionnaire has been translated into several languages, e.g. Turkish [4], Czech [5], German [6]. The Hungarian adaptation of the 49-item Oral Health Impact Profile (OHIP-49-H) questionnaire has recently been developed and validated [2]. Although several studies were aimed at the development and validation of the OHIP-49 questionnaire, only few of them evaluated the influence of clinical factors on OHRQoL using OHIP-49 as a tool [4, 7–9].

Prosthetic replacement of missing teeth, especially with fixed partial dentures, has been shown to exert a beneficial effect on OHRQoL [7, 8]. However, published results on the impact of replacing old dentures to a new one on self-reported OHRQoL are somewhat controversial, in particular with respect of the type of the old and new dentures. Moreover, there are no comprehensive data available on the effects of objective demographic factors, such as age and gender and of objective clinical factors determined by the dental surgeon, such as cariological and periodontal conditions influencing patients’ perception on OHRQoL.

The purpose of the present study was to assess self-reported OHRQoL among patients requiring oral rehabilitation and to evaluate changes in OHRQoL 1 month as well as 6–12 months following prosthetic treatment using OHIP-49-H. The effect of age, gender, oral health indicators and denture types before and after treatment were evaluated.

## Methods

### Subjects

The study was conducted among Hungarian adults undergoing oral rehabilitation. Data were collected from September 2010 to December 2011 in the Faculty of Dentistry of the University of Debrecen and in 13 related outpatient clinics from urban and rural communities. Inclusion criteria were as follows: age ≥ 18 years, need for prosthetic replacement, ability to complete the OHIP-49-H questionnaire without assistance. The Institutional Review Board of the University of Debrecen approved the project. All participants signed an informed consent form.

### Questionnaire

The instrument of the present investigation (OHIP-49-H) was the Hungarian adaptation of the original 49-items version of the self-administered OHIP-49 questionnaire developed by Slade and Spencer [2]. Items were grouped into seven subdomains and respondents were required to answer the questions according to the frequency of the problems using a 5-point Likert scale (0, never; 1, hardly ever; 2, occasionally; 3, fairly often; and 4, very often) according to the proposal by Slade and Spencer based on the assumptions made by Locker et al. [10–12]. OHRQoL of the respondents was characterized by the sum of the subdomain scores, the total OHIP-49-H score according to the recommendation of John et al. [13]. All subdomain effects were statistically significant and not too different in magnitude and correlated highly and significantly with the total score (data not shown). A lower total score represented less, a higher score more impaired OHRQoL.

An additional non-comparative question concerning patients’ oral health condition was added to OHIP-49-H in order to confirm the construct validity of the method:

1. How do you rate your own oral health at the moment?

The oral health condition was registered by a five-grade scale (0, excellent; 1, very good; 2, good; 3, fair; and 4, poor) using lower scores for better status. Evaluating the first 203 questionnaires, associations between the above non-comparative question and total OHIP-49-H scores in different phases of care were evaluated using Spearman’s rank correlation.

### Data collection

The OHIP-49-H questionnaires were completed by 389 consecutive prosthetic patients (214 patients from the university outpatient clinic and 175 patients from community outpatient clinics in urban and in rural environments) before dental treatment without assistance on first admission (T0-phase), and were examined and treated by the working group of authors. The corresponding patients were selected sequentially with their admission without any special selection process. The same questionnaire was completed again by 235 patients (176 patients from the university outpatient clinic and 59 patients from community outpatient clinics in urban and in rural environments) 1 month (T1-phase) and 92 patients (60 patients from the university outpatient clinic and 32 patients from community outpatient clinics in urban and in rural environments) 6–12 months after treatment (T2-phase), respectively [9].

Oral examination and treatment: Cariological and periodontal condition of the participants were documented. DMF-T index was calculated as published previously by Baume [14]. Community Periodontal Index (CPI) was calculated to describe the periodontal status [15].

At baseline (T0-phase) patients were categorized into three groups according to the types of dentures: those having fixed dentures or wore natural dentition (FPD); those with partial removable dentures (RPD); and those with complete dentures (CD). In cases, when someone wore different dentures in the upper and lower jaws or wore a combined denture in the same jaw the most debilitating type of denture had been considered [9]. According to the types of prosthetic interventions nine subgroups were distinguished (Table 1). Ten patients who were complete denture wearers at baseline and were rehabilitated with fixed or partial removable dentures, received implants.

**Table 1:** Application of different types of prosthetic rehabilitation

type of prosthetic intervention (T0 → T1)	number of patients
fixed partial denture → fixed partial denture	71
partial removable denture → partial removable denture	27
complete denture → complete denture	31
fixed partial denture → partial removable denture	41
fixed partial denture → complete denture	31
partial removable denture → fixed partial denture	5
partial removable denture → complete denture	19
complete denture → fixed partial denture	3
complete denture → partial removable denture	7
total	235

### Data analysis

The median interquartile range (IQR) values of the total OHIP-49-H score were calculated for T0-, T1- and T2-phases. We established the correlation between total OHIP-49-H scores of the follow-ups and the minimally important difference (MID), that defines the smallest change in a treatment outcome that a patient would identify as important [16]. Evaluating the first 203 questionnaires, reliability of the OHIP-49-H was checked by Cronbach’s statistics [17]. Spearman correlation test was computed in order to assess the association between non-comparative question concerning patients’ oral health condition and OHIP-49-H scores. Treatment-associated changes in OHRQoL were evaluated by Mann–Whitney U test. Relationships between clinical parameters and changes of OHIP-49-H scores were investigated by stepwise multivariate logistic regression analysis comparing the T0- and T1-phases of the study, after dichotomizing the outcomes according to the observed median values. We used stepwise multivariate logistic regression analysis when we had one nominal variable and two or more measurement variables, and we wanted to know how the measurement variables affected the nominal variable and to understand the functional relationship between the independent variables and the dependent variable, to try to understand the likely cause of the change in the dependent variable. The level of significance was p < 0.05.

## Results

OHIP-49-H was applied in a study group of 389 consecutive dental patients on first visit (T0-phase). Two hundred forty three patients were female (62.5 %) and 146 (37.5 %) patients were male. The mean ± SD age of the respondents was 55.7 ± 13.1 years. The second (T1) and the third (T2) questionnaires were completed by 235 and 92 patients, respectively. Response rate was 60.4 % at T1-phase and 23.7 % at T2-phase.

The sample size at T0- and T1-phases, but not at T2-phase, made it possible to compare major baseline (T0) characteristics of patients who were followed from T0 to T1-phases and those who were lost from follow-up after T0-phase. However, the two groups of patients (i.e. that were followed-up until at least T1-phase and those who were lost from follow-up) did not exhibit any significant differences between major characteristics (age, gender, denture type) confirming the representativeness of the sample (Table 2).

**Table 2:** Characteristics of patients who were followed from T0 to T1-phases and those who were lost from follow-up after T0-phase

	followed in T1	lost from T1
No. of patients	225	164
age		
mean	56.25	55.12
SE	0.84	1.06
p*	0.399	
sex		
male proportion	39.72 %	34.29 %
SE	3.34 %	3.59 %
p**	0.270	
denture		
natural/fixed	33.71 %	31.31 %
partial denture	17.71 %	16.82 %
complete denture	48.57 %	51.87 %
p**	0.809	
* t-test		
** chi-square test		

Evaluating the first 203 questionnaires, the fairly high and narrow range of Cronbach’s alpha values (from 0.81 to 0.93) demonstrated the excellent reliability and internal consistency of the questionnaire (data not shown). There was highly significant correlation between total OHIP-49-H scores and non-comparative question concerning patients’ oral health condition at baseline (T0-phase) (Table 3).

**Table 3:** Association between self-rated OHRQoL and OHIP-49-H scores in different phases of care by Spearman’s rank correlation. All p-values for the correlation coefficients were less than 0.001

	T0	T1	T2
	rho	rho	rho
functional limitations	0.423	0.660	0.447
physical pain	0.301	0.550	0.365
psychological discomfort	0.370	0.579	0.407
physical disability	0.349	0.557	0.402
psychological disability	0.295	0.600	0.468
social disability	0.258	0.539	0.315
handicap	0.280	0.377	0.357
total OHIP-49-H	0.391	0.651	0.477

CPI assessed at T0-phase showed, that 35 % of patients had severe (CPI4), 43 % had moderate (CPI3), and 14 % had mild periodontitis (CPI2) before dental rehabilitation. Three percent of participants had gingivitis (CPI1) and 5 % had healthy periodontium (CPI0). The median DMF-T index was 24 in the sample at T0-phase.

**Fig. 1:**
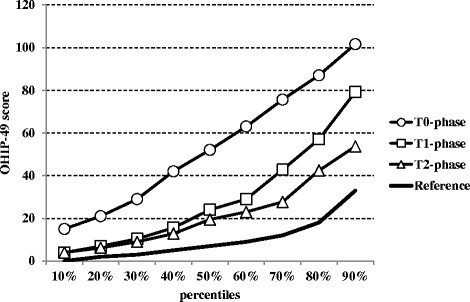
Changes of self-rated OHRQoL as evaluated by the percentile total OHIP-49-H scores and their components on admission (T0-phase: Ο), 1 month (T1-phase: Φ0Φ0) and 6–12 months (T2-phase: Δ) after the dental treatments. Mann–Whitney test was used for statistical comparison. The reference values (continuous black line) represent the Hungarian general population [8]

The median total scores at T0-, T1- and T2-phases and the reference value of the Hungarian general population before any treatment [9] are shown in Fig. 1. The shift of scores towards a range indicating an improved oral health and related satisfaction in course of oral rehabilitation was significant for the total OHIP-49-H. The median total OHIP-49-H score, indicating seriously impaired OHRQoL, was 52 on first visit and it decreased significantly by 1 month to 24 (*p* < 0.001), and decreased further to 20 (*p* < 0.055) 6–12 months after treatment (Fig. 1). From baseline (T0-phase) to first follow-up (T1-phase) change in total OHIP-49-H score was more than the MID. From first to second follow-up (T2-phase) total OHIP-49-H score change was less than the MID.

The sample size at T0- and T1-phases, but not at T2-phase, made it possible to apply multivariate logistic regression analysis to determine the impact of clinical factors with possible influence on therapeutic results and self-assessed OHRQoL. Age was inversely related to the decrease at total OHIP-49-H score (*p* = 0.020) and in handicap score (*p* = 0.005). In case of psychological discomfort (*p* = 0.029) and physical disability (*p* = 0.014) OHRQoL, improvement among females was greater than among males. The limited number of patients in the nine different subgroups of prosthetic interventions did not allow a meaningful statistical evaluation of the effect of age and gender in the outcomes of the individual subgroups. DMF-T value didn’t exert a significant effect on any of the outcome parameters. The CPI value assessed at T0-phase significantly influenced changes in OHRQoL at T1-phase (*p* = 0.011). The higher the CPI value was at T0-phase, the more pronounced OHIP-49-H score reduction was observed at T1-phase, resulting in a more expressive improvement in OHRQoL (Table 4).

**Table 4:** Factors exerting significant effects on changes in total and subdomain OHIP-49-H scores between baseline (T0-phase) and first follow-up (T1-phase). Stepwise logistic regression analysis

	Factors with significant influence	adjusted OR	p
total OHIP-49-H			
	AGE	0.97	0.020
	t0-CPI	1.45	0.011
OHIP-49-H subdomain scores			
physical pain	RPD-- > CD	3.49	0.041
psychological discomfort	GENDER (female/male)	2.06	0.029
	FPD-- > CD	3.31	0.008
	RPD-- > CD	9.99	0.003
physical disability	GENDER (female/male)	2.12	0.014
psychological disability	RPD-- > RPD	0.30	0.014
social disability	RPD-- > RPD	0.33	0.022
	FPD-- > RPD	0.34	0.011
handicap	AGE	0.96	0.005
	CD-- > CD	3.47	0.021

Effects of prosthetic interventions on OHRQoL were evaluated by comparing OHIP-49-H scores before and one month after the insertion of new prostheses (Table 4). The type of prosthetic interventions did not exert a significant effect on total OHIP-49-H score, suggesting that the improvement in OHRQoL is independent from the type of denture applied. Some subdomain scores however, exhibited significant changes in association with the type of prosthetic intervention. Replacement of old CD with a new one resulted in a significant reduction in the handicap item score (*p* = 0.021). Application of a new CD to change old RPD or old FPD, resulted in significant, higher than median improvements in physical pain reduction (*p* = 0.041) and in psychological discomfort minimization item scores (*p* = 0.003) for RPD replacement, and in the psychological discomfort minimization item score for FPD (*p* < 0.008). The improvement of social disability achieved by replacing old FPD with RPD (*p* = 0.011) or with a new one (*p* = 0.022) proved to be also significant.

## Discussion

The purpose of the present study was to investigate self-assessed OHRQoL among 389 patients requiring prosthetic rehabilitation and to determine the rate of improvement 1 month as well as 6–12 months following treatment using the validated Hungarian version of the OHIP-49 questionnaire. Previous Hungarian investigations applied the instrument on a low number of clinical prosthetic patients and did not examine the effects of investigator-derived, objective clinical parameters such as age, gender, oral health indicators and type of dentures before and after the intervention with a potential influence on self-reported OHRQoL.

Processing the results of the first 203 questionnaires excellent reliability and internal consistency of the OHIP-49-H was proven by the high and narrow range of Cronbach’s alpha values (0.81-0.93). The above result confirmed the results of a previous study aimed at the development of the Hungarian version of the OHIP-49 questionnaire [2]. Due to the larger sample-size and the representative nature of the investigated patient population the range of Cronbach’s alpha values were narrower in the present study than found by Szentpétery et al. (0.71-0.96) [2]. Good construct validity of the questionnaire was confirmed by high correlation coefficient values between non-comparative questions concerning patients’ oral health condition and OHIP-49-H scores.

Self-rated OHRQoL of the study population requiring prosthodontic intervention was considerably worse than that of the Hungarian general population before any treatment. Median total OHIP-49-H of patients at T0 was 52 (range 25–83) whereas the same value, characterizing 1059 randomly selected persons was 7 (range 0–37) [8]. According to the Mann–Whitney U test, OHRQoL significantly improved in case of the total OHIP-49-H scores between baseline and the first follow up. The short term effect of the therapy was significant, exceeding MID, comparing the results obtained at the T0- and T1-phases. Between the first and second follow-ups we have observed a marginal further improvement in the OHRQoL characterized by a near-significant decrease in total OHIP-49-H less than MID. The above finding confirmed preliminary results obtained by the John et al. According to their findings OHRQoL of 76 % of their patients improved rapidly 1 month following treatment and it underwent further significant but moderate improvement 6–12 months after therapy in 90 % of patients [9].

According to the demographical and T0 phase clinical status, the treatment was more effective in the respect of OHIP-49-H score improvement among females (than among males), among younger (than among more aged), and among patients with more serious CPI assessed at T0. DMF-T status did not influence significantly OHIP-49-H scores. According to the investigation of Ng and Leung, individuals with periodontal disease had lower OHRQoL compared with patients having healthy periodontal conditions [18]. In our study patients with higher initial CPI experienced a more expressed improvement in self-reported OHRQoL at the later check-up phases of the study.

The type of dentures may influence OHRQoL. Removable dentures (RPD, CD) have frequently been associated with complaints due to inappropriate design and manufacture. More favorable objective results can be achieved with fixed dentures. The correlation however, between self-reported OHRQoL and the type of original and new dentures seems to be somewhat controversial. Therefore, we have investigated the effect of the type of prosthetic intervention on OHRQoL in course of rehabilitation 1 month (T1-phase) and 6–12 months (T2-phase) after the first visit. The first QoL study conducted on 107 prosthetic patients in the University of Halle found similar outcome to ours after dental rehabilitation [9]. Using OHIP-53-G John et al. evaluated the improvement of QoL in three different denture groups. The fastest and highest QoL development was observed in case of patients treated with FPD, while the least favorable outcome was found among patients treated with RPD [9]. A group of investigators from the University of Pecs has reported that fixed dentures were superior in the respect of patients’ satisfaction [8, 19]. Two years later the same group reported results of a follow-up study which were still considered preliminary in nature because of the restricted sample size involving 63 prostodontic patients [7]. Kende et al. confirmed that patients having their own teeth or FPD presented the lowest OHIP-49-H, whereas RPD proved to be the most and CD a moderately debilitating denture type. OHRQoL improved rapidly within 1 month after treatment and it underwent further but moderate improvement within the following 6 to 12 months after treatment in subjects with fixed, removable and complete dentures. The most impressive improvement was observed in patients treated with FPD [7]. In our study we did not find a significant change in total OHIP-49-H score related to the type of prosthetic intervention. However, results of our study may become intuitive from the clinical point of view in the perspective using MID of OHIP-49-H. Because of the effectivity of the prosthodontic intervention, regardless of its type, total OHIP-49-H score exhibited a decrease more than MID between baseline and first follow-up. In contrast, in the period between the first and the second follow-up, where patients did not receive treatment any more but adaptation to the new denture was still going on, further improvement was marginal, less than MID of OHIP-49-H.

## Conclusions

Here we presented representative data about self-assessed OHRQoL of patients requiring prosthetic treatment from Hungary using OHIP-49-H questionnaire. The results demonstrated that the restoration of oral health was associated with an improvement in patients’ OHRQoL. According to the demographical and T0 phase clinical status, the treatment was more effective in the respect of OHIP-49-H score improvement among females (than among males), among younger (than among more aged), and among patients with more serious CPI assessed at T0.The type of prosthetic interventions did not exert a significant effect on total OHIP-49-H score, suggesting that the improvement in OHRQoL is independent from the type of denture applied.
